# Supplementary material for: Chromothripsis during telomere crisis is independent of NHEJ, and consistent with a replicative origin
Source: Genome Res. 2019 May;29(5):737–49. doi: 10.1101/gr.240705.118 (PMC6499312; doi:10.1101/gr.240705.118)
Supplement: Supplemental Material [file supp_gr.240705.118_Supplemental_file_1.zip › contigs/annotated_contigs/DB51/contig.5.DB51_length_785_mean_cov_5.87898089172.docx]

**DB51_length_785_mean_cov_5.87898089172**

```
AAACAGAGATGGCTTGACCACAGTGACCAGCCTGCACCCTAATGTCCTCCAGGACTTTCCCAGAAATTCA|CG|GGAAATGTAACTCAG
 >chr18:68506673-68506745 + E=2e-31                                    >chr18:67127850-
TTTAAGGCTTTTCTGTGCTTTCCTCAAACATAGTACCATATTATTTTGCACGTGCACACACACACAGAAACAAGACAGGTTCAAAAGAC
67128248 - E=1e-224
CAAGTATGAAGCAGAAAGTGTTGCTGCTCTGACAGTTAAAAAGTGGGCATGATCGAAGTCAAGATGAGACTGCAGGAAAGGCAGAGGTA

GGCTTTTAAATGATGCTTATGAAGAATGAAAGAGGGCAATACAATTATAAGGCAAGAAGCCACTATTTTAACACGTCTTTATGAGTAGA

AAAGTAGTATATTCAGTGGTTTAGTTGCAAAATAATAAAAACACATAACTGATTATATAAATTTACCAAACCAATGAGTTTGCCAGATA

TCAACAATTTGTTACAATTTCTTCA|CT|AATCTGGCAAGAAAATCACAGAAGATGATAATG|CTGAAAGGAAT|TTAAGTATACCAGA
                            >chr18:68489469-68489502 - E=5e-05 p=3e-02  >chr18:684954
TAATTTAGGAACGTTATTAGAAAATAACTGCCTTCATGTTT|TG|GCAGGTCAGAAACTAAATGAAATAGAAGAGGCTTGAAAATAAGC
58-68495515 - E=2e-22                     >chr18:67128373-67128589 - E=4e-115
AAAAATAAACTCATAAAACATAAAAGTAATTCTGGGAAGCATTTCTGAGTTCCAGATAAGATTGCAAGTGCTTCTAATAAAATACATTT

ATAAGTTTGTGAGTGTGTTTCTGAGAGAGAGACAATGTGTGTGTGTGTTTCTGAAGTATATGTGTTCATCAGAACCAAAAA
```
